# The Cyclopædia of Anatomy and Physiology

**Published:** 1839-04-01

**Authors:** 


### ON THE FUNCTIONS AND DISEASES OF THE EAR.

I. THE CYCLOPÆDIA OF ANATOMY AND PHYSIOLOGY. Edited by *Robert B. Todd*, M.D. F.R.S. &c. &c. Part XV. London, January, 1839. Price five shillings.

II. A TREATISE ON THE STRUCTURE, ECONOMY, AND DISEASES OF THE EAR; BEING THE ESSAY FOR WHICH THE FOTHERGILLIAN GOLD MEDAL WAS AWARDED BY THE MEDICAL SOCIETY OF LONDON. By *George Pilcher*, Lecturer on Anatomy and Surgery at the Theatre of Anatomy and Medicine, Webb-street, Borough; and Senior Surgeon to the Surrey Dispensary. London, 1838, pp. 324.

### I. THE FUNCTIONS OF THE EAR.

THE appearance of the present Part of the Cyclopædia of Anatomy has been materially delayed, but the editor promises the publication of the next part in a month, and the completion of the work as early as possible. The best way, we fancy, for the public to get the work rapidly concluded, is to patronize it.

The Part before us contains—

Hearing, Organ of, concluded, by T. Wharton Jones, Esq.
Hearing, by Dr. Todd.
Heart, Normal Anatomy, by Dr. J. Reid.
Heart, on the Arrangement of the Fibres of the, by H. Searle, Esq.
Heart, Abnormal Anatomy, by Dr. Todd.
Heat, Animal, by Dr. Edwards.

We can speak in very favourable terms of the execution of all the articles. The work is really a very valuable one, and deserves encouragement from the scientific part of the profession—more than, we fear, it gets. Yet other works, of inferior merit, have received more. If individuals cannot afford to purchase it, all medical libraries and book societies should have it.

We shall select the article Hearing, by Dr. Todd, for an analytical notice. We do so, not because it is better than the others, but because the majority of medical men know little of the physiology of the ear, as most that has been learnt on the subject is of recent date.

We need not dilate on the importance of hearing. But a few words on sound may not be misapplied.

Sound is the result of an impulse of any kind made on the organ of hearing. Usually, the impulse is conveyed by the air. The body by which the sound is produced, denominated by Professor Wheatstone *a phonic*, occasions in the surrounding air vibrations or oscillations, corresponding in number and extent to those which exist in itself; and these vibrations or oscillations being propagated to the organ of hearing, give rise to the sensation. It would be idle to multiply instances of this. The sound of a cannon fired is as good as any.

But the sound may be communicated directly through the bones of the cranium, or it may arise from direct contact of a solid with the tympanum. Every body who has had a tooth drawn must have been struck with the sound occasioned by the extraction; and the beating of the carotids in cases of nervousness or of cardiac hypertrophy, is a familiar instance of the sound conveyed by the solid structure of the cranium. A fine probe introduced carefully through the meatus externus, and made to impinge upon the membrana tympani, however gently, will occasion the sensation of sound.

Any irregular impulse communicated to the air produces a noise, in contradistinction to a *musical sound*. This latter results from a succession of impulses, which occur at exactly equal intervals of time, and which are exactly similar in duration and intensity. When these impulses succeed each other with great rapidity, the sound appears continuous, in consequence of the duration of the impression upon the auditory nerve. The frequency of repetition necessary for the production of a continued sound from single impulses is, according to Sir J. Herschel, probably not less than sixteen times in a second, though the limit would appear to differ in different ears. In this respect hearing is analogous to sight. A rapid succession of images upon the retina produces the idea of uninterrupted colour or light. The schoolboy's amusement of twirling a burning stick in the air is a common example of this.

We distinguish in musical sounds, 1, the *pitch*; 2, the *intensity* or *loud-*

*ness;* 3, the *quality.* The pitch of the sound depends on the rapidity with which the vibrations succeed each other, and any two sounds produced by the same number of vibrations or impulses in the same time are said to be *in unison.* The loudness or intensity depends upon the violence and extent of the primitive impulse. The quality is supposed by Sir J. Herschel to depend on the greater or less abruptness of the impulses, or generally, on the law which regulates the excursions of the molecules of air originally set in motion.

Different media convey sound with different rates of velocity. In air at the temperature of 62° Fahr. sound travels at the rate of 1125 feet in a second, or 1090 feet in a second in dry air at the freezing temperature.

This velocity is independent of the pitch and quality of sound. Distance does not destroy the harmony of a rapid piece of music played by a band. Biot caused several tunes to be played on a flute at the end of a pipe 3120 feet long, and found that they could be distinctly heard without the slightest derangement.

"Neither is the velocity of sound affected by an increase of density in the air. It is, however, greater in warm than in cold air in consequence of the greater elasticity of the former. In the different gases much variety has been observed in the velocity of sound; through carbonic gas the rate of the velocity is said to be one-third slower than ordinary, but through hydrogen gas, which is twelve times more elastic than common air, the speed exceeds the usual rate three and a half times. A more striking difference is as regards the intensity of sound or the impression it is capable of producing on our organs of hearing. This varies considerably with the increase or diminution in the density of the transmitting gas. By means of a piece of clock-work, which caused a hammer to strike at regular intervals, the conducting power of the gas could be estimated, the clock-work being placed in a glass receiver filled with the gas. It was thus that Priestly ascertained that in hydrogen the sound was scarcely louder than in vacuo; in carbonic acid and in oxygen it was somewhat louder than in air." 566.

Water can transmit sound, or we may well suppose that God would not have given ears to fish. M. Colladon, by means of a tin cylinder three yards long, and eight inches in diameter, closed at its lower end but open to the air above, plunged vertically in the water, was enabled to hear the sound of a bell at the distance of about nine miles, and from numerous observations he concluded that the velocity of sound in water at about 46° Fahr. was equal to 4708 feet in the second.

Solids are still better conductors of sound, and those which are homogeneous, hard, and elastic, are the best. A conclusive experiment, by Herhold and Rahn, is related by Chladni:—a metallic wire 600 feet long was stretched horizontally, and at one end a plate of sonorous metal was attached; when the plate was slightly struck, a person at the opposite end, holding the wire in his teeth, heard at every blow two distinct sounds, the first transmitted almost simultaneously by the metal, the other arriving later through the air. Biot, with the assistance of Messrs. Boulard and Malus, concluded the velocity of sound in cast iron at the temperature 51° Fahr. to be 11,090 feet in a second.

Sound, like light, admits of reflexion, the angle of which equals the angle of incidence. Echos are such reflexions, and, the sound being reflected once or oftener, the echo is correspondingly single or repeated.

"The phenomena of echos illustrate beautifully the analogy between sound and light. Thus, the reflexion of sound from concave and convex surfaces takes place exactly as in the case of light: if a reflecting surface be concave towards an auditor, the sounds reflected from its several points will converge towards him, exactly as reflected rays of light do; and he will receive a sound more intense than if the surface were plane, and the more so the nearer it approaches to a sphere concentric with himself; the contrary is the case if the echoing surface be convex. If the echo of a sound excited at one station be required to be heard most intensely at another, the two stations ought to be *conjugate foci* of the reflecting surface, i. e. such that if the reflecting surface were polished, rays of light diverging from one would be made after reflexion to converge to the other. Hence, if a vault be in the form of a hollow ellipsoid of revolution, and a speaker be placed in one focus, his words will be heard by an auditor in the other, as if his ears were close to the other's lips. The same will hold good if the vault be composed of two segments of paraboloids having a common axis, and their concavities turned towards each other; only in this case sounds excited in the focus of one segment will be collected in the focus of the other after two reflexions.

The most favourable circumstances for the production of a distinct echo from plane surfaces is when the auditor is placed between two such exactly half-way. In this situation the sounds reverberated from both will reach him at the same instant, and reinforce each other: if nearer to one surface than the other, the one will reach him sooner than the other, and the echo will be double and confused." 567.

*Functions of the Ear.*—Dr. Todd examines, in succession, the offices or functions of the internal ear—the middle ear—and the nerves.

1. *Of the Internal Ear.*—It is probable that the *vestibule* forms the most essential part of the apparatus of hearing, from the fact that it is met with in every class of animals in which such an apparatus can be found. Here is the seat of the principal expansion of the auditory nerve upon the saccule and common sinus, which floating in the perilymph communicate, through the medium of that fluid, with the membrane of the fenestra ovalis, and consequently with the air contained in the tympanum. Any vibrations or oscillations then excited in the membrane of the fenestra ovalis, cannot fail to affect the perilymph to a proportional extent, and through it the membranous vestibule. In the simple ear of crustaceous, of cephalopods, and of the lowest cyclostomous fishes, the sonorous impressions are conveyed to the vestibular cavity through its solid parietes; and even in the higher organized fishes the labyrinth, enclosed in the solid cranium, receives through it the impulses of sound, and has no communication with the external air.

Dr. Todd touches on the part played by the *otolithes*, or calcareous concretions, and by the *otokonies*, or calcareous dust, found in the sacculus vestibuli of the ears of cephalopods and fishes. But though, no doubt, their vibration assists in producing the sensation of sound, their precise mode of action does not seem to us to be elucidated.

*a.* In man, and in animals provided with an external ear and tympanum, as well as labyrinth, the sonorous vibrations are apparently communicated in two ways by the meatus externus and tympanum to the vestibule and semicircular canals—and by the bones of the head direct to the auditory nerve.

"Sounds proceeding from external bodies, as Weber observes, are conveyed in the former way; but the oscillations of one's own voice, although they in part find their way by the external passage, are chiefly conducted by the cranial bones; and, as Professor Wheatstone has remarked, those sounds are best heard which are articulated most in the mouth, and with that cavity least open, as *e*, *ou*, *te*, *kew*. Closing both ears by firmly pressing the hands upon them, one's own voice is not heard less distinctly, but on the contrary more loud and clear than when both ears are left open; and if only one ear be closed, the voice is heard more distinctly and louder in that ear than in the open one." 368.

There can be no doubt of the communicability and communication of sounds, and, amongst others, of the hearer's voice, through the solid parts of the head. But, however it may be with others, *we* cannot hear our own voice better with our ears closed than with them open. We conceive that this is a bit of a refinement on the part of Weber, and we suspect, too, that if Dr. Todd will plug his ears effectually and articulate either syllables or words, he will find them rendered less distinct, not more so. At all events, this is the case with ourselves, and it is what one would anticipate. But to proceed:—

"One or two experiments with the tuning-fork show not only that the cranial bones do conduct, but also that sounds, inaudible or imperfectly audible through the meatus externus, may be distinctly heard when the sounding body is brought into contact with a bone of the cranium or face. When the tuning-fork is put into vibration by striking it against any solid body, if held near the external ear its vibrations are heard distinctly, but let the handle be applied to the teeth or to the superior maxilla, and the sound appears much louder; or if the fork be held near the ear until the sound has almost died away, and then its handle be applied to the superior maxilla or the teeth, the sound seems greatly to revive and continues for a considerable period, the handle being kept in contact with the bone.

When the conducting stem of the sounding tuning-fork is placed on any part of the head, if both ears be closed by being covered with the hands, a considerable augmentation of the sound will be observed.* If the sounding-fork be kept in contact with the head for a short time, both ears remaining open, and then one ear be closed, the sound will be greatly augmented in the closed ear, and will appear to be heard exclusively by it. This experiment is more striking if the stem of the tuning-fork be applied to the mastoid process on one side: when both ears remain open, the sound seems to be heard chiefly by the ear in the vicinity of which the stem is placed, but when the opposite ear is closed, it appears as if the sound were transferred from the open to closed ear; and if the ear be alternately opened and closed, the sound will alternately appear to be transferred from the one to the other. Similar phenomena may be observed if both ears are closed on the first application of the tuning-fork. The sound is at first heard in the adjacent ear, and either remains in it or is transferred to the opposite one, according as the former remains closed or is opened. Mr. Wheatstone adds that if the meatus and concha of one ear be filled with water, the sound from the tuning-fork will be referred to the cavity containing the water in the same way as when it contained air and was closed by the hand.

These phenomena afford obvious examples of the communication of sound through the bones of the head. The augmentation of the sound in the closed ear appears to result, as Mr. Wheatstone explains, from the reciprocation of the vibration, by the air contained within the closed cavity, and this explanation is confirmed by the fact, that when the meatus is closed by a fibrous substance, such as wool, no increase is obtained." 568.

* "These experiments were first suggested by Professor Wheatstone.—See his experiments on audition in the Journal of the Royal Institution for July 1827."

These facts, if they be facts, are certainly curious. But if they possess no surer basis than the statement that we hear our own voice best when the ears are stopped, they must be received with a little reservation.

If we pronounce such sounds as *e* or *kew*, with one ear open and one closed, we are told that they are heard more distinctly and loudly by the closed ear than by the open one. We regret that it is not our good fortune to find it so. On the contrary, the sound is less distinct and less loud, though there is a sort of rough, subdued reverberation distinguishable in the closed ear. This, we should say, rather deadens the sound, than augments it. As we have no opportunity, at present, of making experiments with the tuning-fork, we say no more upon this subject.

*Functions of the Cochlea.*—It would, of course, be desirable to ascertain these, if possible. But it is not easy to do so.

*a.* The observations and experiments of Weber render it very probable that the cochlea is that part of the labyrinth which is more particularly suited to appreciate sounds communicated through the solid case of the head, or, to use his words, that sounds propagated through the bones of the head are heard specially by the cochlea, but that sounds conducted through the external meatus are perceived by the membranous vestibule and semicircular canals more easily than by the cochlea. The following considerations favour these views.

It is an admitted fact in acoustics that sounds are most perfectly conducted by substances of uniform elasticity, and that when propagated from air or water to a solid, or from a solid to air or water, they are conducted much less completely. Now, inasmuch as the cochlea may be regarded as part and parcel of the cranial bones, the sounds which are propagated by these bones would reach the nervous expansion in that portion of the labyrinth by the most direct route; whereas, to affect the remaining parts of the labyrinth, the sound must be conducted from the bone through the perilymph to the membranous vestibule and semicircular canals. Moreover, when it is considered that the cochlear nerves are disposed in a radiated manner in the lamina spiralis, it will appear evident that the oscillations propagated to the petrous portion of the temporal bone must exert a direct influence on the cochlear portion of the auditory nerve.

Such is the account which Dr. Todd gives of the views of Weber. They may be admitted to be plausible, but that is all; and, as Dr. Todd remarks, they throw no light on the peculiar form and mechanism of the cochlea.

*b.* Autenrieth and Kerner believed the cochlea to be that part of the auditory apparatus by which we perceive what the French call the "timbre" of sounds; that quality, namely, which depends on the nature of the material of which the sounding body is constituted, as well as on its form and size, and in some degree on the manner in which sound is elicited from it; and they considered it the office of the vestibule to convey to the sensorium the pitch and strength of sounds. Their opinion as to the function of the cochlea was founded on some experiments as to the extent to which certain of the lower animals were affected by particular instruments of music: the results obtained from these experiments, when taken in connexion with certain differences in the form and other characteristics of the cochlea in those animals, led these authors to the conclusion that "those animals alone seemed to perceive a difference of the 'timbre' of sounds of pretty uniform pitch and loudness, in whom the cochlea was very long, and projected considerably into the cavity of the tympanum, and was not much concealed by the surrounding bony substance. Thus it appeared that a dog (the cochlea of dogs being longer than that of cats), upon hearing a certain note of the clarionet, set up a howl, but seemed in no way affected at hearing the same note from the flute or violin; but the cat continued undisturbed, although a variety of instruments was sounded in her hearing. A rabbit (in which the cochlea is prominent) ran away at the note C elicited from a glass tumbler or from a string, but remained still when the same note was sounded even more loudly by the flute."

We quite agree with Dr. Todd in his opinion of the unsatisfactory nature of these experiments, and of the flimsy texture of the theory they support. It would be useless arguing a question of so unsubstantial a character.

*c.* Whatever the precise use of the cochlea may be, it is found in an advanced condition of the organ of hearing, and, therefore, implies much perfection of the sense.

*Functions of the Common Sinus and Semicircular Canals.*—The connexion of the fenestra ovalis and membrana tympani, through the medium of the ossicula, makes it natural to suppose that the semicircular canals and common sinus must be especially affected by the sounds which are conveyed through the meatus externus. An experiment of Weber illustrates the relation of the perilymph to the membranous labyrinth, and shows that an impulse upon the membrana tympani is capable of affecting it. In some birds, the falcon for example, the semicircular canals are so large, that the membranous canals may be easily seen. If in such a bird one osseous semicircular canal be opened by a small opening, care being taken not to injure the membranous canal, and then we press the membrane of the tympanum inwards, at each compression we observe the water contained in the bony canal to flow out with a jerk. He therefore concludes that the sonorous undulations conveyed by the cranial bones are communicated more immediately to the nerve of the cochlea, but those conveyed by the external meatus to the nerve of the vestibule.

"The semicircular canals are remarkable for the constancy of their number, and of their relative position with respect to each other, in all animals in whom they are found. They exist in almost all fishes, and in all the other vertebrate classes, and in these they are never less than three in number, two of which are always placed vertically, and one horizontally. The opinion that the arrangement of these canals has reference to conveying the sensation of the direction of sounds, I find expressed by Autenrieth and Kerner in the paper already referred to. 'In no animal,' they say, 'are these canals ever more or fewer on each side than three, which are so situated that they correspond to the three dimensions of a cube, its length, breadth, and depth, and that every sound arriving in one of these three directions will always strike one canal at right angles to its axis, and another in its length. The position of these canals is likewise such, that the corresponding canals of opposite sides cannot be parallel, and that therefore any sound which strikes the head in any given direction, affects the semicircular canal of one side much more than the corresponding one of the opposite side, whereby it may be determined whether the sound coming in a straight line (from west to east for example), has really moved from west to east, or from east to west.' They state, that in animals in whom the semicircular canals are highly developed, the power of distinguishing the direction of sounds is marked to a proportionate degree. Thus in the mole, the development of these canals is very considerable, and from a simple experiment it appears that this animal readily distinguishes the direction of sounds. A mole was introduced into a wide but flat vessel filled with earth, in which he was allowed to burrow, and it was found that the mole could be made to move about by sounding an instrument outside the vessel; if the instrument were sounded on one side, the animal would always immediately turn to the other." 570.

This looks too pat to be true. We think it was John Bell, who said that experimenters were the greatest liars on earth. Without going so far as that, it must be owned that they do often say odd things. They resemble that mythological monster which could hear excellently with one ear, but very indifferently with the other. Experimenters have a singular facility in listening to whatever chimes with their fancies, and as obdurate a deafness to what does not. Thus Autenrieth and Kerner had no sort of difficulty in perceiving that a mole "*always and immediately*" turned to the side on which *they* sounded an instrument. But Esser, who repeated the experiment, found no such thing. He "assures" us that the direction of the movements of the mole was *not* influenced by the direction of the tones of the instrument. Some might feel it hard to explain this contradiction on a mere matter of fact. We are old stagers, and feel no difficulty on the subject.

The human semicircular canals greatly exceed in width all others examined by Autenrieth and Kerner, but this excess is more as regards the canals properly so called, but does not apply to the ampullæ. Scarpa had already remarked, that although the canals of oxen and horses were narrower than those of man, the ampullæ were scarcely at all smaller than in the human subject. These observers further remarked, in many animals they examined, an inverse ratio between the width of the ampullæ and that of the canals; that the former were wider in proportion as the latter were narrow. In fine, they conclude, as has been already hinted, that, cæteris paribus, the wider the semicircular canals, the greater the power of distinguishing the direction of sounds.

"Professor Wheatstone advocates the theory that our notions of audible direction depend upon the excitation of those portions of the auditory nerve which belong to the semicircular canals. He conceives that we distinguish best the direction of those sounds which are sufficiently intense to affect the bones of the head, and that it is from the portion which is transmitted through those bones that our perception of the direction is obtained. Thus, we always find it difficult to tell by the ear the position whence the feeble tones of the Œolian harp proceed. The three semicircular canals, then, being situated in planes at right angles with each other, are affected by the sound transmitted through the bones of the head with different degrees of intensity, according to the direction in which the sound is transmitted; for instance, if the sound be transmitted in the plane of any one canal, the nervous matter in that canal will be more strongly acted on than that in either of the other two; or if it be transmitted in the plane intermediate between the planes of this canal and the adjacent one, the relative intensity with which those two canals will be affected, will depend upon the direction of the intermediate plane. The direction suggested to the mind will correspond with the position of the canal upon which the strongest impression has been made."* 570.

Our readers, at least many of them, are probably aware that Flourens has made some very cruel, bloody, and bootless experiments on living animals, to determine the functions of the different parts of the ear. When we reflect how difficult the dissection of the labyrinth is, even when the bone containing it is set in a vice before us, we may infer, without hesitation, the value of vivisections in such a case. We may say with Dr. Todd, that, had M. Flourens never attempted these experiments, physiology would have been none the worse, and our respect for his humanity would have been all the greater.

This is all that our author has to say on the functions of the labyrinth. It must be perceived and cannot but be owned, how vague are the ideas of the offices of its several parts. Plausible conjecture is almost all that we have, and, as experiments on animals are almost prohibited by the nature of the subject, we are compelled to rest on analogy and on pathology. The former scarcely goes far enough—the latter has hitherto not assisted us much. But, as the structure of the ear is more examined in cases of congenital or acquired deafness, we may hope that advances will be made in our acquaintance with the part played by the several portions of the labyrinth.

*Of the Accessory Parts of the Organ.* These are composed of, and Dr. Todd successively examines, the external ear—the tympanum, its membrane, and ossicles—and the Eustachian tube.

*The External Ear.*—This may be considered as made up of the auricle and of the meatus externus.

The auricle is found completely developed only in mammifera, and exists pretty generally throughout the class, though varying much in form. It is said that those animals which are remarkable for the large development of the auricle are almost all timid or nocturnal, and consequently require an acute sense of hearing. In such animals there can be little doubt that the auricle is used as an ear-trumpet, for the purpose of collecting and concentrating sounds. Treviranus thinks that in the lower animals, but *especially in man*, the principal use of the auricle is in determining the direction of sounds. Dr. Todd observes that a remark of Mr. Gough, the author of a highly interesting paper in the Manchester Memoirs " on the method of judging by the ear of the position of sonorous bodies," offers a strong argument against this notion. He observes that, whatever may be the direction of a sound in the open air, as soon as it enters the auditory passage, it is compelled to follow the course of that duct until it reaches the apparatus in which the sense

* "Dr. Young thought that the semicircular canals seemed very capable of assisting in the estimation of the acuteness or pitch of a sound by receiving its impression at their opposite ends, and occasioning a recurrence of similar effects at different points of their length, according to the different character of the sound; while the greater or less pressure of the stapes must serve to moderate the tension of the fluid within the vestibule, which serves to convey the impression. The cochlea seems to be pretty evidently a micrometer of sound.—See Med. Lit. p. 98."

of hearing resides. We will not pretend to say that in man, the auricle does contribute to the discrimination of the direction of sounds, but we do not think Mr. Gough's objection so fatal to the hypothesis as it seems to Dr. Todd. When the motion of the auricle is voluntary, it is very possible that the muscular action becomes a sort of empirical source of knowledge respecting the direction of sound. Certain actions are associated with such motions of the auricle as render the sound more distinct. That distinctness leads, by experience, to a knowledge of direction. This is analogous with the process by which we become sensible of the position of our limbs, the weight of bodies, perhaps even the distance of objects. Of course what we have observed applies only to animals who have voluntary motions of the auricle. It is difficult to suppose that the auricle contributes much to the knowledge of the direction of sound in man.

The experiments of Savart are calculated to throw light on the functions of the meatus externus as well as of the membrana tympani.

If a thin membrane, such as very fine paper, is carefully stretched horizontally over the mouth of a glass, or of a small delft basin, and if a thin layer of sand is spread on this, and a glass thrown into vibration by a violin bow is held at a little distance from it; that the paper immediately begins to vibrate is shewn by the motions excited in the sand, the particles of which arrange themselves into figures, which are sometimes perfectly regular, and which form with so much rapidity that the eye can scarcely follow " the circumstances which accompany the transformation of the thin layer of sand into a greater or less number of lines of repose." It is clear from these data, that vibrations are communicated from one body through the air to another. The next step was to shew that the membrana tympani is so affected by the aerial undulations excited by a sonorous substance. This Savart proved to be the fact.

The temporal bone having been separated, he sawed away the osseous meatus so as to expose the membrane on a level with the rest of the bone, and when it was sufficiently dry, he covered it with a thin layer of sand. A vibrating glass held parallel and very near to the surface of the membrane, occasioned a slight movement of the grains of sand, but, owing to the slight extent and the shape of the membrane, it was impossible to determine the existence of any nodal line. In a second experiment, the cavity of the tympanum was opened, so as to expose the ossicles of the ear and their muscles; and it was observed that when the internus mallei muscle acted and rendered the membrane tense, it was much more difficult to produce manifest movements in the grains of sand; thus affording much reason to suppose the tensor tympani muscle is analogous in its use to the iris, and destined to preserve the organ from too strong impressions.

Dr. Todd goes on to state :—

"In imitation of the mechanism by which the tension of the membrana tympani is effected, and with a view to determine more decisively the effects produced by variation of the tension of that membrane, Savart constructed a conical tube, with its apex truncated and covered by a layer of very thin paper, which was glued to the edge of the opening. A little wooden lever, introduced through an opening in the side of the tube, and resting on the lower margin of this opening as a fulcrum, was used to vary the tension of the membrane, one of its extremities being applied to the under surface of the membrane. It is evident that, by depressing the extremity of the lever that was external to the tube, the inner one would be raised, and thus the membrane stretched to a greater or less degree according to the force used ; on the other hand, by elevating the outer extremity, the inner one was separated from the membrane, which was accordingly restored to its original tension. This little lever was employed in imitation of the handle of the malleus, which under the influence of its muscles causes the variation in the tension of the membrana tympani. The artificial tympanic membrane then having been covered with a layer of sand, it was found that, under the influence of a vibrating glass, used as in the former experiments, a manifest difference was produced in the movements of the grains of sand, by increasing the tension of the paper ; the greater the tension, the less the height to which the grains of sand were raised; and these movements were most extensive when the lever was withdrawn from contact, and the membrane left to itself.

From these experiments Savart concludes that the membrana tympani may be considered as a body thrown into vibration by the air, and always executing vibrations equal in number to those of the sonorous body which gave rise to the oscillations of the air. But what is the condition of the ossicles of the tympanum whilst the membrane is thus in vibration ? The result of the following experiment affords a clue to the answer of this question. To a membrane stretched over a vessel, a piece of wood uniform in thickness is attached, so that the adherent part shall extend from the circumference to the centre of the membrane, while the free portion may project beyond the circumference. When a vibrating glass is brought near this membrane, very regular figures are produced, which however are modified by the presence of the piece of wood, and the vibrations of the membrane are communicated to the piece of wood,on which likewise regular figures may be produced. The more extensive the membrane, the longer and thicker may be the piece of wood in which it can excite oscillations, and Savart states that, with membranes of a considerable diameter, he has produced regular vibrations in rods of glass of large dimensions. The oscillations of the piece of wood are much more distinct when the adherent portion is thinned down, by which it seems, as it were, more completely identified with the membrane, and consequently the oscillations of this latter are communicated directly to the thinned portion of the wood, and thence extended to the thick portion : sand spread upon it will exhibit active movements, and will produce very distinct nodal lines. Thus it may be inferred that the malleus participates in the oscillations of the tympanic membrane; and these vibrations are propagated to the incus and stapes, and thus to the membrane of the fenestra ovalis." 573.

The chain of ossicles forms, as has generally been supposed, a conductor of vibrations, but the malleus, in addition, regulates the tension of the membrana tympani. Dr. Todd has carefully repeated the experiments of Savart, with precisely similar results.

Savart wished to ascertain how far the external ear and auditory canal increase the vibrations of the membrane.

He formed, writes Dr. Todd, a conical tube of pasteboard, with a very wide mouth at its base, the opening at the smaller end being closed by a thin paper stretched over it and glued to the margins of the opening. This tube is placed resting on its base, the membrane being upwards and perfectly horizontal, so that a layer of sand may be spread over it. When a vibrating glass is brought near and parallel to the upper surface of this membrane, it immediately begins to vibrate, and the grains of sand are tossed about, but raised but very slightly from the surface. If, however, the vibrating glass be placed near the base or the wide and open extremity of the tube, the vibrations of the membrane will be found to be much more manifest, and the excursions of the grains of sand so considerable, that they are often raised to a height of three or four centimetres; so that there is a manifest difference in the influence produced upon the membrane by the sonorous undulations excited in the air according as they impinge upon the external surface of the membrane or upon that which is turned towards the interior of the tube. This phenomenon, Savart adds, may depend upon two causes, namely, upon the concentration of the sonorous undulations by the tube, or upon the communication of motion to the parietes of the tube, which again would communicate it to the membrane. With a view to ascertain which of these causes was the effective one, a second conical tube, open at both ends, was held with its narrow extremity a little above and corresponding to the narrow extremity of the former one, but so that there was no contact between them. If now the glass is made to vibrate successively at the large orifices of the two tubes, it will be found that, when placed at the orifice of the tube to which the membrane is attached, the oscillations of that membrane are considerably greater than when the aerial undulations reach it through the other tube. Whence it may be inferred that in all probability the external ear and auditory canal have, besides any influence they may exert in modifying the movements of the particles of the air, the additional function of presenting a large surface to the aerial undulations, consequently to enter into vibration under their influence, and thus to contribute to increase the excursions of the vibrating parts of the membrane with which they are immediately in contact; the auricle, by the variety of the direction and the inclination of its surfaces to one another, can always present to the air a certain number of parts, whose direction is normal (at right angles with) to that of the molecular movement of that fluid.

A familiar instance of the use of the external ear in collecting and directing the sonorous undulations, may be seen in the assistance often given to hearing, by placing the hollow hand behind the auricle and drawing the latter forward. This deaf people frequently do. And the loss of the auricle is said to be followed by dullness of hearing, particularly in those animals in which the osseous meatus is wanting.

*Action of the Stapedius.*—The contraction of the stapedius muscle causes the base of the stapes to compress the membrane of the fenestra ovalis to a greater or less extent, so that the degree of tension of that membrane depends on the condition of this muscle. Compression exerted upon the membrane of the fenestra ovalis extends to the perilymph and through it is propagated to the membrane of the fenestra rotunda, and in this way the same apparatus which regulates the tension of the membrane of the fenestra ovalis performs that office for that of the fenestra rotunda. Savart has invented a neat apparatus for the illustration of the manner in which this may take place. We have not space, however, for the description of it.

It appears, continues Dr. Todd, from the anatomy of the ear, "that the only muscles which have been satisfactorily demonstrated are tensors of the tympanum; and that at whatever extremity of the chain of ossicles muscular effort be first exerted, a corresponding effect will be produced at the other; that when the stapedius muscle acts, the malleus is thrown into a position favourable to the tension of the membrana tympani, and, on the other hand, the contraction of the internus mallei depresses the stapes, and consequently increases the tension of the membranes of the two fenestræ. The cessation of muscular action restores all three membranes to their original laxity, nor does it appear that they admit of any further degree of relaxation through the influence of any vital process. The incus forms a bond of connexion between the two other bones, and its motions depend entirely upon theirs in consequence of its articulation with both, while from the fixedness of its connexion with the mastoid cells, as well as its intermediate position, and its not having any muscles inserted into it, it is obvious that its motions must be much more limited than those of the other bones. Its use seems to be to complete the chain in such a way, that by reason of its double articulation with the malleus on the one hand and the stapes on the other, the tension of the tympanic membranes may be regulated without any sudden or violent motion, which could scarcely be avoided were the conductor between the membranes of the tympanum and fenestra ovalis one piece of bone."

Savart offers the following conjectures of the use of the tympanum, its membrane and its ossicles. If the membranes of the fenestra, he says, had been in immediate contact with the atmosphere, their elastic state would have been constantly undergoing changes, under the influence of the vicissitudes of temperature of the air, a circumstance which would, in all probability, impair the power of the organ in detecting differences of sounds. He presumes therefore that the membrana tympani prevents this contact of the atmosphere with the membranes of the labyrinth, and that the cavity of the tympanum and the mastoid cells form a kind of receptacle in which the air, which finds its way into the tympanum through the Eustachian tube, acquires the constant temperature of the body, and establishes in front of the openings of the labyrinth a sort of atmosphere proper to themselves, the temperature of which does not vary.

*Inaudibility of Certain Sounds.*—Dr. Wollaston observed, that "an ear which would be considered as perfect with regard to the generality of sounds may at the same time be completely insensible to such as are at one or the other extremity of the scale of musical notes, the hearing or not hearing of which seems to depend wholly on the pitch or frequency of vibration constituting the note, and not upon the intensity or loudness of the noise." And we owe to this distinguished man the knowledge of the interesting fact, that an insensibility of the ear to low sounds may be artificially induced, by exhausting the cavity of the tympanum to a great degree. This may be effected by forcibly attempting to take breath by expansion of the chest, the mouth and nose being kept shut; after one or two attempts, the pressure of the external air is strongly felt upon the membrana tympani, which is thus from the external pressure thrown into a state of considerable tension. An ear in this state becomes insensible to grave tones, without losing in any degree the perception of more acute ones. This induced defective state of the ear, from exhaustion of the tympanum, may even be preserved for some time without the continued effort of inspiration and without even stopping the breath, and may in an instant be removed by the act of swallowing. "If I strike the table before me with the end of my finger," continues Dr. Wollaston, "the whole board sounds with a deep dull note. If I strike it with my nail, there is also at the same time a sharp sound produced by quicker vibrations of parts around the point of contact. When the ear is exhausted, it hears only the latter sound, without perceiving in any degree the deeper note of the whole table. In the same manner, in listening to the sound of a carriage, the deeper rumbling noise of the body is no longer heard by an exhausted ear; but the rattle of a chain or screw remains at least as audible as before exhaustion."

The same condition of the tympanum is produced by the sudden increase of external pressure. This is felt on descending in the diving bell, which, according to Wollaston occasions closure of the Eustachian tube. Dr. Todd descended in the bell at the Polytechnic Institution. The first effect, he tells us, of the pressure on the tympanic membrane was the production of a crackling noise, which was immediately succeeded by a painful sense of pressure in both ears: but this is immediately relieved by the act of swallowing; it soon, however recurs, and may be in a like manner relieved. Grave tones were least distinctly heard.

"In such cases then it would appear that from the strong compression exerted on the membrana tympani, that membrane cannot vibrate in unison with tones which result from a small number of vibrations. On the other hand we may infer, from Dr. Wollaston's observations, that 'human hearing, in general, is more confined than is generally supposed with regard to its perception of very acute sounds, and has probably in every instance some definite limit at no great distance beyond the sounds ordinarily heard.' The ordinary range of human hearing comprised between the lowest notes of the organ and the highest known cry of insects, includes, according to Wollaston, more than nine octaves, the whole of which are distinctly perceptible by most ears. Dr. Wollaston has, however, related some cases in which the range was much less, and limited as regards the perception of high notes; in one case, the sense of hearing terminated at a note four octaves above the middle E of the piano-forte; this note he appeared to hear rather imperfectly, but the F above it was inaudible, although his hearing in other respects was as perfect as that of ordinary ears; another case was that of a lady who could never hear the chirping of the gryllus campestris; and in a third case the limit was so low that the chirping of the common house-sparrow could not be heard. Dr. Wollaston supposes that inability to hear the piercing squeak of a bat is not very rare, as he met with several instances of persons not aware of such a sound." 575.

*Integrity of the Membrana Tympani not essential to Hearing.*—It is unnecessary to dwell on this. The membrane has been destroyed, or punctured, without loss of hearing, sometimes with advantage to it.

*The Eustachian Tube.*—This has evidently a double office. It carries air into the tympanum—and it affords an outlet for the escape of such sonorous undulations as do not impinge upon the labyrinthic wall of the tympanum, which, were there no such communication with the external air, would cause an echo, and in this respect it performs a function similar to that of the mastoid cells.

*Functions of the Nerves.*—Not much can be said on this subject. The portio mollis of the seventh is evidently the nerve of hearing; and the muscular apparatus of the tympanic ossicles receives its nerves partly from the facial and partly from the otic ganglion, thus exhibiting an analogous arrangement to that of the muscular structure of the iris. The offices of the chorda tympani, and of the tympanic anastomosis are far too conjectural to be dwelt on.

Dr. Todd concludes with the following brief summary of what is known, or, rather, supposed, respecting the functions of the several parts of the organ of hearing.

1. The *vestibule* is the essential part of the organ. It detects the presence and intensity of sound, and especially of those sounds conveyed through the external ear and tympanum.

2. The *cochlea,* lying in immediate connection with the bone, receives those sounds which are propagated through the bones of the head. According to Kerner it is the medium of the perception of the *timbre* or quality of sounds.

3. Of the function of the *semicircular canals* we know nothing. That they aid in forming a judgment of the direction of sounds is conjectured by Autenrieth and Kerner, and more recently by Wheatstone.

4. The *tympanum* and its membrane render the internal ear independent of atmospheric vicissitudes, and the former affords a non-reciprocating cavity for the free vibration of the latter, as well as of the chain of ossicles.

5. The *chain of ossicles* acts as a conductor of vibrations from the membrana tympani to the fenestra ovalis, and under the influence of the muscles regulates the tension of the membrana tympani, as well as the membrane of the fenestra rotunda, so as to protect the ear against the effects of sounds of great intensity.

6. The *external ear* and *meatus* are conductors of vibrations; the former in some degree collects them as a hearing-trumpet would do, and probably assists in enabling us to judge of the direction of sounds.

We have given this article at considerable length for two reasons. In the first place, the majority of medical men are probably very imperfectly acquainted with the functions of the auditory apparatus. In the second place, the diseases of the ear are beginning to interest the profession, and will probably, ere long, be snatched from the hands of the ill-informed people who now monopolize the treatment of them. We are anxious to assist in effecting this desirable object, and we have latterly seized every opportunity of diffusing information on the subject. This we shall still continue to do, and it will not be our fault if our readers do not become acquainted with what has been or will be made out in reference to acoustic medicine.

### II. THE DISEASES OF THE EAR.

The present treatise by a lecturer on anatomy and surgery gives promise of the diseases of the ear being soon generally studied; and, although we lately reviewed at some length a translation of Dr. Kramer's work, showing the present state of knowledge on the diseases of the ear, yet we shall not hesitate to devote a few pages to the essay of Mr. Pilcher, and thus further the excellent object which the London Medical Society seems to have had in view—that of calling the attention of the profession to the subject.

The treatise is divided into two parts—the first devoted to the Anatomy of the Ear and Physiology of Hearing, and the second to the Diseases of the Ear. In the first we find collected, a clear digest of the anatomy and functions of the organ of hearing, and of its development in the animal gradation, exceedingly useful, as conveying correct information on this division of the subject, preparatory to the efficient study of the diseases of the organ, and in a very accessible form. As we have already dwelt on the functions of the ear, we need not revert to them now.

To the second division, devoted to the abnormal condition of the various structures entering into the formation of the ear, Mr. Pilcher directs attention, as the most important part of the work. The malformations which have been observed, and such slight treatment as their nature generally permits, form an introduction to this second part. In reference to this it is observed that, "as different portions of the organ, at different periods of formation, bear an analogy more or less striking to similar parts in the lower animals, so it is easy to trace in their malformations a correspondence to the permanent structure of the inferior classes."

The only malformations of the auricle which require surgical aid, Mr. Pilcher defines to be the absence of the auricle, and the closure of the opening; "the former appearing to lessen but slightly the power of hearing," and the deficiency can only be supplied by an artificial ear. The second malformation admits of remedy in two ways. "The overlapping of the tragus and antitragus, if prejudicial to hearing, may be readily removed by the knife; or if it be of small extent, dilatation by the gentle pressure of a tube or a tent may be sufficient."

Contraction of the meatus auditorius externus is next noticed, to be treated by dilatation. In cases of "obliteration of auditory-canal" (non formation rather ?) an operation is recommended, under certain conditions, for creating one by a trocar. No case however is quoted in illustration, and it seems of hazardous nature and promising but little success. When only a fold of skin passes across, it is easy then to conceive that the bistoury or trocar might effect a permanent opening and much improve the condition of the patient.

Many other malformations are enumerated, affecting the middle and internal ear, but as they are beyond the surgeon's cut and consequently lead to few practical inferences, they are chiefly interesting as showing how frequently deafness may occur from structural alterations over which we can exercise no control.

We may observe, in reference to these cases generally, and even where an operation requiring both nicety and boldness is proposed, that in no instance has any case been brought forward to prove how far experience has shown it practicable with success or otherwise.

On coming to the consideration of the effects of disease on the various structures of the organ of hearing, we were glad to find that Mr. Pilcher at the outset states, that in its pathology the ear does not differ from other structures. In Dr. Kramer's work—and the same observation applies generally to treatises on the ear—its diseases are treated in a manner curiously contrived to render that complicated which is simple, and to give the impression that the diseases and the structures of the ear are unlike all others to which the human frame is liable. Whether such a conclusion was meant to be inculcated by their authors we will not to venture affirm, but that such is the direct tendency of the mode in which the subject is generally handled, admits of no dispute. A conclusion at least as injurious as it is erroneous. Such views too naturally flow from those who devote the whole of their time and attention to the treatment and consideration of any one organ or disease; effects common to all, are insensibly resolved into peculiarities in the minds of such exclusive practitioners. Hence it follows, that the diseases of the ear, the eye, &c. will always be best understood and treated by those who practice the profession generally. Not that we would imply that they are to be thoroughly comprehended or skilfully treated without particular study—on the contrary, an exclusive study of their varieties, and the various operative means required for their relief is absolutely required; but in the same manner as the eye has been very generally studied of late years as a branch of surgical practice, and not as the "one thing only needful," should the diseases of the ear be superadded to the usual course of surgical education.

The introductory paragraph so entirely coincides with the view we took of this subject in the review of Dr. Kramer's work, that we shall extract it as curiously confirmatory.

"In its pathology the ear does not differ from other structures. From this general conclusion, perhaps, may be excepted inspissation of wax in the meatus, the exact analogue of that secretion not being elsewhere met with. Inflammation, therefore, is the affection to which, for the most part, this organ is obnoxious; and as this disease is modified in its progress, in its symptoms, and in its effects, by the structures which it attacks,—by the local predisposition of the organ affected,—by the general constitution of the individual,—by the causes which may excite it,—by the external circumstances, or conditions which accompany it,—so have the affections of the ear been arranged under various heads, nearly all of which may be reduced to inflammation and its consequences. As in all other organs, of which the seat of function is in an expanded nerve, the ear is likewise subject to an anæsthetic affection, dependent on some hidden morbid condition, and occasionally on inflammation, either of the acoustic nerve, or of those parts of the brain in connection with the organ. Again, in consequence of the vicinity of the ear to the brain, it often participates in the diseases and accidents of that organ; and the reverse is frequently observed of disease extending from the ear to the brain, or the skull. Lastly the accidental introduction of foreign bodies into the meatus; the rupture by a blow or otherwise of the membrana tympani, have been esteemed peculiar to the ear; but perhaps hardly with justice, as nearly similar accidents are met with in the nasal and visual organs." 155.

Mr. Pilcher takes a somewhat similar view to that which we expressed respecting Dr. Kramer's arrangement of diseases—somewhat confused and very unnecessarily complicated—presenting many distinctions without differences, he fell into the very error he so gravely and severely censured in all other writers, ancient and modern. Thus

"Kramer has arranged the diseases of the auditory passage under the heads of—1st. Erysipelatous Inflammation;—2ndly. Inflammation of the Glandular Structure of the Meatus;—3rdly. Inflammation of the cellular Tissue; 4thly. Inflammation of the Periosteum. This division is useful to a certain extent, and every surgeon should bear in mind that each structure of the canal may be in certain cases the chief seat of inflammation; but that acute otitis is likely to continue exclusively in any one structure during its entire progress is more than doubtful. In most cases, perhaps, the disease commences in the glandular apparatus, but passes so rapidly to the cellular membrane, that the surgeon may not have had an opportunity of witnessing the affection in its original seat." 172.

Some interesting cases are given, illustrative of the fatal facility with which inflammation of the structures of the ear extends to the brain and its membranes, affording, moreover, additional reasons in support of our argument, that these are diseases which ought not to be the object of exclusive treatment.

In reference to the greatest improvement in auricular surgery, for which "entre nous soit dit" we are indebted to the post-master of Versailles, and not to either surgeon or aurist, catheterism of the Eustachian tube, Mr. Pilcher suggests an improved form with a whalebone stilet, which he thinks better than either wire or catgut, and as he speaks from personal experience, having often employed it in the cases he details, its merits should be carefully tested and the instrument adopted instead of Itard and Kramer's, if the result be favourable.

The perusal of this work has afforded us much pleasure, for if we have discovered but little essentially original matter, we are not the less sensible of the complete and comprehensive manner in which the whole subject has been treated. If not dictated by such large experience in this peculiar class of diseases as may have furnished notes for the Berlin Doctor, it is nevertheless superior in the simplicity of arrangement, and the careful exclusion of all unnecessary complications or wire-drawn distinctions, which may by ingenuity be made clear upon paper, but can never be in like manner read in the patient's symptoms or appearances. A work was wanted to place the whole subject within the grasp of all surgeons who choose to devote some little exclusive or particular study to the diseases of the ear and their treatment, and this has fairly and well supplied the deficiency.

---

